# The Role of Adjuvant Radiotherapy in the Treatment of Breast Cancer

**DOI:** 10.3390/curroncol31030090

**Published:** 2024-02-24

**Authors:** Iveta Kolářová, Bohuslav Melichar, Igor Sirák, Jaroslav Vaňásek, Jiří Petera, Kateřina Horáčková, Denisa Pohanková, Filip Ďatelinka, Zuzana Šinkorová, Milan Vošmik

**Affiliations:** 1 Department of Oncology and Radiotherapy, Faculty of Medicine in Hradec Králové, University Hospital Hradec Králové, Charles University, 500 05 Hradec Králové, Czech Republic; iveta.kolarova@fnhk.cz (I.K.); jiri.petera@fnhk.cz (J.P.); denisa.pohankova@fnhk.cz (D.P.); filip.datelinka@fnhk.cz (F.Ď.); milan.vosmik@fnhk.cz (M.V.); 2 Faculty of Health Studies, Pardubice University, 532 10 Pardubice, Czech Republic; jaravanasek@seznam.cz (J.V.); katerina.horackova@upce.cz (K.H.); 3 Department of Oncology, Faculty of Medicine and Dentistry, University Hospital Olomouc, Palacky University, 779 00 Olomouc, Czech Republic; bohuslav.melichar@fnol.cz; 4 Oncology Centre, Multiscan, 532 03 Pardubice, Czech Republic; 5 Department of Radiobiology, Faculty of Military Health Sciences, University of Defence, 500 01 Hradec Králové, Czech Republic; zuzana.sinkorova@unob.cz

**Keywords:** breast cancer, adjuvant radiotherapy, whole breast irradiation, mastectomy, breast-conserving surgery, regional node irradiation, neoadjuvant chemotherapy

## Abstract

The role of postmastectomy radiotherapy and regional nodal irradiation after radical mastectomy is defined in high-risk patients with locally advanced tumors, positive margins, and unfavorable biology. The benefit of postmastectomy radiotherapy in intermediate-risk patients (T3N0 tumors) remains a matter of controversy. It has been demonstrated that radiotherapy after breast-conserving surgery lowers the locoregional recurrence rate compared with surgery alone and improves the overall survival rate. In patients with four or more positive lymph nodes or extracapsular extension, regional lymph node irradiation is indicated regardless of the surgery type (breast-conserving surgery or mastectomy). Despite the consensus that patients with more than three positive lymph nodes should be treated with radiotherapy, there is controversy regarding the recommendations for patients with one to three involved lymph nodes. In patients with N0 disease with negative findings on axillary surgery, there is a trend to administer regional lymph node irradiation in patients with a high risk of recurrence. In patients treated with neoadjuvant systemic therapy and mastectomy, adjuvant radiotherapy should be administered in cases of clinical stage III and/or ≥ypN1. In patients treated with neoadjuvant systemic therapy and breast-conserving surgery, postoperative radiotherapy is indicated irrespective of pathological response.

## 1. Introduction

Adjuvant radiotherapy (RT) after mastectomy or breast-conserving surgery may result in two principal potential benefits, i.e., lower risk of locoregional recurrence, and a reduction in breast cancer mortality risks and overall mortality [1,2,3].

**Figure 1:**
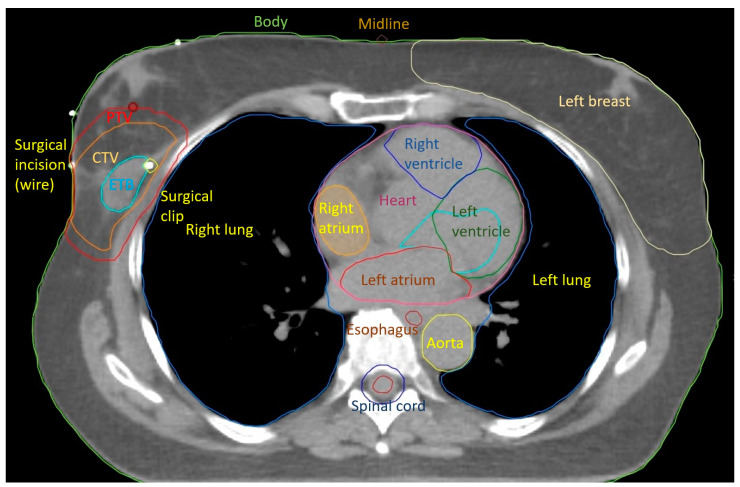
3D computed tomography deep learning-based organ at risk auto-contouring (Siemens Healthineers AG, Erlangen, Germany) with manual target volume (CTV, PTV) delineation. ETB = Estimated Tumor Bed.

**Figure 2:**
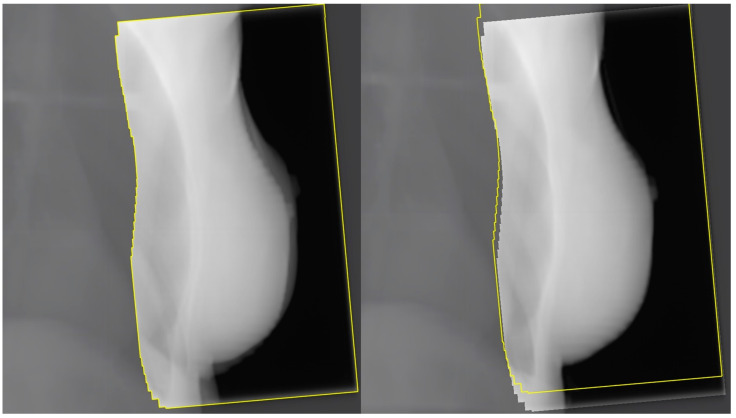
2D megavoltage electronic portal imaging (EPID) as a daily IGRT online setup of breast carcinoma radiotherapy: before (**left**) and after (**right**) alignment.

**Figure 3:**
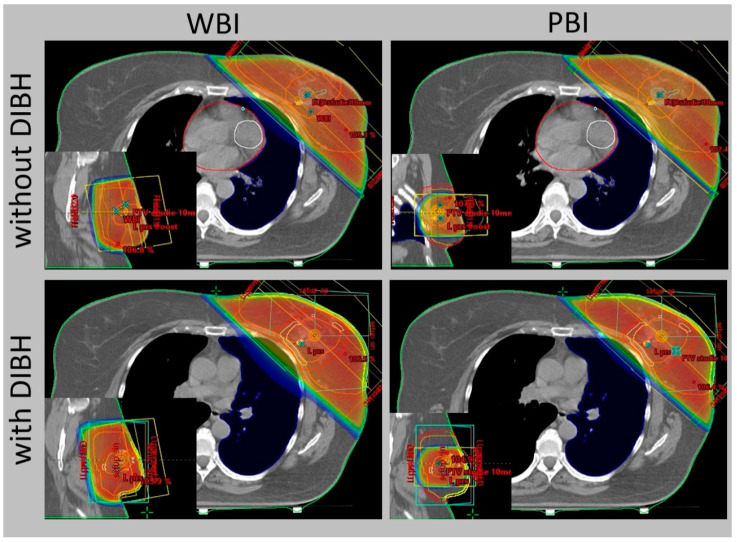
External-beam whole breast irradiation (WBI) and partial breast irradiation (PBI) with and without deep inspiration breath hold (DIBH)—comparison of the same target volume CT slice.

The progress in radiotherapy is based mostly on the use of new technologies. Currently, external-beam radiation is performed using linear accelerators after computed tomography-based imaging (Figure 1) and 3-dimensional planning using sophisticated technologies, including intensity-modulated radiation therapy (IMRT). The control of the accuracy of the dose delivery can be increased with image-guided radiation therapy (IGRT, Figure 2). The dose to the heart can be substantially decreased by delivering radiation in deep inspiration (Figure 3) or prone position [4,5].

Advances in radiobiology have resulted in an improved understanding of mechanisms of acute and late toxicity leading to a deeper understanding of the biological effects of different fractionation regimens. Owing to this progress, hypofractionated radiotherapy that significantly reduces the treatment duration has been increasingly used in recent years [6].

## 2. Radiotherapy after Radical Mastectomy

Modified radical mastectomy is the most common procedure after primary (neoadjuvant) systemic therapy in patients with locally advanced breast cancer. The rationale for using modified radical mastectomy also includes planning reconstructive surgery with breast replacement, avoidance of adjuvant radiation therapy, and the presence of mutations associated with a high risk of second primary breast cancer. Adjuvant radiotherapy is indicated after mastectomy not only in patients with locally advanced tumors but also based on the presence of risk factors. In selected patients, radiation therapy after mastectomy improves local control as well as overall survival [7].

The disease recurrence pattern was analyzed in high-risk breast cancer patients after mastectomy treated with systemic therapy with or without radiotherapy. In patients treated with radiotherapy, the incidence of locoregional recurrence and distant metastases was lower compared to patients who had no radiation. The 18-year probability of any first breast cancer event was 73% vs. 59% (*p* < 0.001) after no RT and RT, respectively (relative risk [RR], 0.68; 95% CI, 0.63 to 0.75) [2].

**High-risk patients.** The role of PMRT (postmastectomy radiotherapy) and regional nodal irradiation after radical mastectomy is defined in patients with locally advanced tumors—T3, T4, (T3N0 controversial), positive margins, gross extracapsular extension, four or more affected nodes, or grade 3—given the fact that PMRT lowers the risk of locoregional relapse and improves breast cancer-specific and overall survival rates by 4–5% [1].

**Intermediate-risk patients.** The benefit of postmastectomy radiotherapy in medium-risk patients (T3N0 tumors) remains a matter of dispute. The role of PMRT was studied in a meta-analysis of National Surgical Adjuvant Breast and Bowel Project (NSABP) trials. In 313 breast cancer patients with tumors of ≥5 cm and no lymph node involvement, the incidence of locoregional failure as a first event was low leading to the conclusion that PMRT should not be routinely used in this patient population [8,9,10].

In a meta-analysis that included 8 135 patients randomized in 22 trials between 1964 and 1986, between the irradiation of the chest wall and regional lymph nodes after mastectomy and axillary surgery or no radiotherapy, no additional effect of radiotherapy on locoregional recurrence was noted in 700 patients with negative lymph node findings after axillary dissection [11].

On the other hand, in an analysis of 2535 patients from the SEER database treated between 2000 and 2010 with modified radical mastectomy for T3N0M0 tumors, PMRT was associated with a significant improvement in both cancer-specific survival and OS, leading to the conclusion that PMRT should be strongly considered in this patient population. Other risk factors including age, tumor grade, lymphovascular invasion (LVI), and margin status may also be considered [10].

**Low-risk patients.** Radiotherapy is not routinely recommended for patients after radical mastectomy with T1-2N0 tumors because of the low risk of local recurrence (1–2%) [11]. Nevertheless, adjuvant radiotherapy may be indicated in selected patients who would otherwise be considered low risk, e.g., patients with close/positive margins on mastectomy, patients aged 35 or younger, and patients harboring tumors with LVI and/or grade 3 [12].

Although some studies indicate improved outcomes after PMRT in all patients with positive lymph nodes, some trials demonstrate a low locoregional recurrence rate, even in the absence of radiotherapy in patients with T1 or T2 tumors and one to three involved lymph nodes. In many patients, the probability of locoregional failure is so low that the risk of toxicity associated with the treatment prevails. However, PMRT should be strongly considered in patients with one to three positive lymph nodes and grade 3, or extracapsular extension [13].

## 3. Radiotherapy after Breast-Conserving Surgery

Residual microscopic disease that may give rise to recurrence may be present in up to 40% of patients after surgical resection. Holland et al. reported that in 43% of cases of apparently unifocal carcinoma, tumor cell nests localized > 2 cm from the primary tumor were present [14].

This observation represents the rationale for whole breast irradiation (WBI) aiming at the eradication of residual disease. It has been demonstrated that WBI after breast-conserving surgery not only lowers the locoregional recurrence rate compared with surgery alone but also improves the overall survival rate at 15 years [1].

The National Surgical Adjuvant Breast and Bowel Project (NSABP) B-06 trial reported a 20-year incidence of ipsilateral recurrence after lumpectomy in 14.3% of patients treated with adjuvant radiation and 39.2% of patients with no adjuvant radiation [15].

An Early Breast Cancer Trialists’ Collaborative Group (EBCTCG) meta-analysis that covered 17 trials that enrolled 10 801 patients confirmed a lower risk of recurrence (from 31% to 15.6%) with adjuvant radiation in patients without lymph node involvement. The 15-year risk of death from breast cancer decreased from 20.5% to 17.2%, demonstrating that adjuvant WBI affects the long-term risk of death from breast cancer. This meta-analysis also indicated that no subgroup of patients defined by age, stage, or hormone receptor status would derive benefit from radiotherapy. Thus, WBI is indicated in the majority of patients after breast-sparing surgery [16].

## 4. Comparison of the Results of Breast-Conserving Surgery and Radical Mastectomy

Several studies address the difference in treatment outcome between radical mastectomy and breast-conserving surgery with subsequent WBI. No difference in overall survival was observed in six large, randomized trials. Consequently, in 1992 the National Cancer Institute put forward a consensus that presented both radical mastectomy and breast-conserving surgery as acceptable standard therapeutic options for patients with operable breast cancer [17].

The Swedish trial published in 2021 compared the efficacy of breast-conserving surgery combined with radiotherapy and radical mastectomy in 48,986 patients who underwent surgery between 2008 and 2017 for T1-2N0-2 breast cancer. The breast cancer-specific and overall survival was significantly better in patients treated with breast-conserving surgery and postoperative radiation compared to radical mastectomy with or without adjuvant radiotherapy. Although this difference remained after correction for tumor characteristics, therapy, demography, comorbid conditions, and socioeconomic status, the effect of selection bias is difficult to exclude. However, these data indicate that breast-conserving surgery with subsequent radiotherapy may improve overall survival [18].

**Figure 4:**
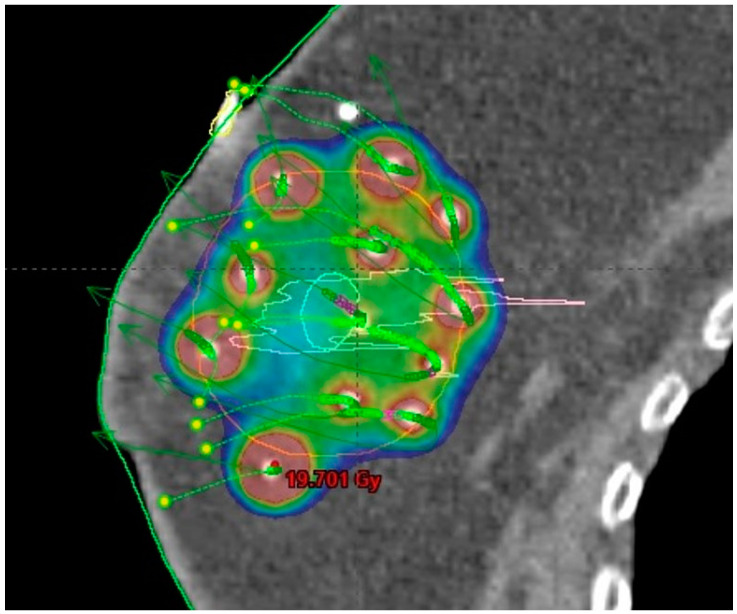
Multicatheter interstitial accelerated partial breast irradiation (APBI): prescribed dose distribution.

The use of breast-conserving surgery is also supported by the availability of the option to modify adjuvant breast irradiation that would further decrease the side effects of therapy compared to WBI. Selected subgroups of patients can be treated with less extensive radiotherapy techniques, including accelerated partial breast irradiation (APBI, Figure 4), partial breast irradiation (PBI, Figure 3), or intraoperative radiotherapy (IORT) [19].

## 5. Radiotherapy after Neoadjuvant Chemotherapy

Neoadjuvant systemic therapy is being increasingly used in the treatment of breast cancer, particularly in patients with HER2-positive and triple-negative tumors. A study evaluating locoregional control in patients treated with breast-conserving surgery after neoadjuvant systemic therapy found after multivariate analysis that increased risk of locoregional relapse was associated with the triple-negative subtype, clinical III stage, and an absence of pathological complete response [20].

A pooled analysis of nine prospective trials of neoadjuvant chemotherapy that included 10,075 patients with a median follow-up of 67 months evaluated the predictors of locoregional recurrence risk. The site of the first relapse was locoregional in 9.5% of patients, distant in 14.5%, and both locoregional and distant in 1.7%. Younger age, clinically positive lymph node, grade 3 tumor, absence of pathological complete response, and triple-negative subtype were independent predictors of locoregional relapse in multivariate analysis. The cumulative 5-year locoregional relapse rate was higher in the absence of pathological complete response compared to patients in whom the pathological complete response has been reached, and this difference reached statistical significance in patients with hormone receptor-positive/HER2-negative, hormone receptor-negative/HER2-positive, and triple-negative tumors, but not in patients with hormone receptor-positive/HER2-positive tumors. Among patients without pathological complete response, the risk of locoregional relapse was higher for patients with hormone receptor-negative/HER2-positive and triple-negative tumors compared to patients with hormone receptor-positive/HER2-negative tumors [21].

In the past, patients treated with neoadjuvant systemic therapy and positive sentinel lymph nodes have been routinely indicated for axillary lymph node dissection. This approach has been recently changed in patients with clinically negative lymph nodes after neoadjuvant therapy. The targeted axillary dissection of a positive lymphatic lymph node marked before neoadjuvant systemic therapy represents a new option in staging [22].

Postoperative WBI is indicated in patients treated with neoadjuvant systemic therapy and breast-conserving surgery irrespective of pathological response. In the case of residual lymph node involvement and patients with clinical stage III disease irrespective of pathological response, regional lymph node irradiation is indicated. In patients treated with neoadjuvant systemic therapy and mastectomy, adjuvant radiotherapy should be administered in cases of clinical stage III and/or ≥ypN1 [23].

The approach to radiotherapy in patients with clinical N1 disease and pathological complete response is a matter of controversy. A meta-analysis investigating the benefit of adjuvant locoregional radiotherapy in patients with clinically positive lymph nodes and pathological complete response treated with mastectomy or breast-conserving surgery demonstrated the benefit of locoregional radiotherapy in lowering locoregional recurrence rate, but no effect on disease-free- or overall survival. However, in clinical practice, the results of this meta-analysis should be interpreted with caution because of the low level of evidence [24].

The results of clinical trials indicate that adjuvant regional lymph node irradiation could be considered in patients with clinical N1 disease and pathological complete response after neoadjuvant systemic therapy. The decision should be based on the evaluation of locoregional relapse risk considering tumor biology, age, and other clinical and pathological characteristics. The optimal management of these patients has yet to be defined (Table 1 and Table 2), and the results of NSABP B-51 are eagerly awaited [25].

## 6. Regional Lymph Node Irradiation and Risk of Serious Toxicity

In a meta-analysis of the Early Breast Cancer Trialists’ Collaborative Group that covers 14 trials, the results of older (1961–1978) trials that included 2178 patients demonstrated an increased risk of death associated with radiotherapy to the regional lymph nodes, probably resulting from the radiation damage to the heart and lungs. On the other hand, regional lymph node irradiation did not affect breast cancer recurrence [Rate ratio (RR) = 0.98, 95% confidence intervals CI 0.85–1.13, *p* = 0.83] or mortality (RR = 1.05, 0.91–1.21, *p* = 0.54), but increased non-breast cancer mortality (RR = 1.44, 1.20–1.73, *p* < 0.0001), leading to a net increase in any death (RR = 1.18, 1.06–1.32, *p* = 0.004).

In newer trials (1989–2003) that included 10 954 patients, regional lymph node irradiation resulted in reduced breast cancer recurrence (RR = 0.86, 95% CI 0.79–0.94, *p* = 0.0006), breast cancer mortality (RR = 0.81, 0.74–0.90, *p* < 0.0001), and overall mortality (RR = 0.86, 0.80–0.93, *p* = 0.0002) [28].

## 7. Internal Mammary and Medial Supraclavicular Lymph Node Chain Irradiation

**Figure 5:**
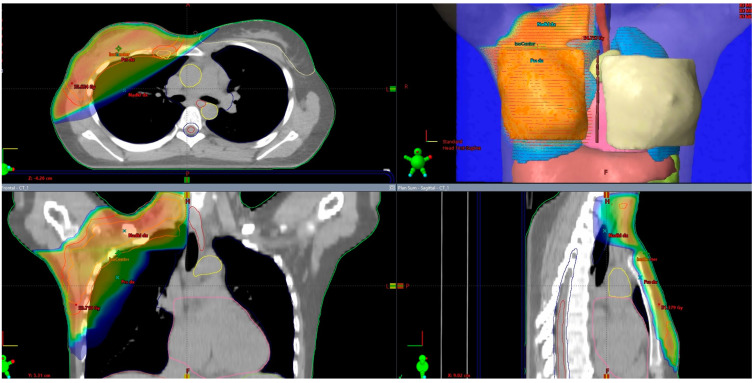
Postmastectomy radiotherapy (PMRT) with axillary, internal mammary, and medial supraclavicular lymph node irradiation.

Internal mammary node irradiation (Figure 5) is another controversial topic. Individual trials did not demonstrate an overall survival advantage of this therapy, and benefits and risks should be carefully weighed. The EORTC 22922/10925 phase 3 trial enrolled 4004 patients younger than 75 years with unilateral, histologically verified stage I to III breast cancer and axillary lymph node involvement or medial tumor location treated with mastectomy or breast-conserving surgery and axillary staging. The patients were randomized at a 1:1 ratio between internal mammary and medial supraclavicular lymph node irradiation with a dose of 50 Gy in 25 fractions or no such treatment. The administration of internal mammary and medial supraclavicular lymph node irradiation resulted in lowering the risk of breast cancer death or any recurrence at 15 years, but no overall survival benefit was observed [29].

A recent meta-analysis of data from five prospective controlled trials with a median follow-up of more than 13 years demonstrated an overall survival benefit of internal mammary node irradiation of 9%, along with improved breast cancer-specific survival, distant metastases-free survival, a lower locoregional recurrence rate, and comparable mortality for comorbid conditions.

However, concerning potential chronic toxicity the benefit of internal mammary node irradiation in all patients is not clear, and the authors concluded that with technological advances leading to lower chronic toxicity the addition of this therapy should be considered in selected patients at higher risk of recurrence. The decision should be individualized for each patient based on clinical risk assessment, age, comorbidity, and availability of high-quality radiation techniques [30].

## 8. The Significance of Axillary Lymph Node Dissection and Radiotherapy in Patients with Clinical N0 Disease and Positive Sentinel Lymph Node Biopsy

The issue of the benefit of completing axillary lymph node dissection (ALND) in patients with clinically negative lymph nodes was addressed by the American College of Surgeons Oncology Group (ACOSOG) Z0011 trial in 891 patients with clinical T1-2N0 disease undergoing lumpectomy with sentinel lymph node biopsy (SLNB) and positive findings in one or two lymph nodes. The protocol stipulated WBI without regional lymph node irradiation in all patients. Patients with more than two lymph nodes involved, extensive extranodal spread, neoadjuvant chemotherapy, and mastectomy were excluded, and 96% of patients were treated with systemic therapy.

The total number of patients was 1900, but the trial was closed prematurely because of low mortality. The primary endpoint, overall survival, was similar in the arms with or without completed ALND. The rates of lymphedema (13% vs. 2% at one year, *p* < 0.0001), wound infection, axillary seroma, and paresthesias were higher in patients treated with ALND compared to SLND. The standard tangential field was prescribed by the protocol, but a high tangential field (defined as equal or less than 2 cm from the humeral head) was used in approximately 50% of cases and irradiation of the supraclavicular lymph nodes was administered in 19% of patients.

The cumulative incidence of nodal recurrences at 10 years was 0.5% in the ALND arm and 1.5% in the SLND alone arm (*p* = 0.28). Ten-year cumulative locoregional recurrence was 6.2% with ALND and 5.3% with SLND alone (*p* = 0.36) [30,31,32,33]. It can be concluded that the completion of axillary lymph node dissection is not mandatory in patients with one or two sentinel lymph node metastases who will subsequently undergo WBI and systemic therapy.

The IBCSG 23-01 trial in patients with one or more micrometastatic (≤2 mm) sentinel lymph nodes and a tumor size ≤5 cm did not demonstrate any difference in recurrence in patients with or without ALND.

The results of the ACOSOG Z0011 and IBCSG 23-01 trials after sentinel node biopsy demonstrated no difference in recurrence rate after axillary lymph node dissection after sentinel node biopsy over the sentinel node biopsy alone in patients with both macroscopic and microscopic metastases [31,32,33,34,35].

The AMAROS trial that compared ALND with radiotherapy for the axilla in patients with positive sentinel lymph node biopsies and negative clinical findings in regional lymph nodes did not report any difference in recurrence rate. The rate of lymphedema was doubled in the ALND arm (28% vs. 14%). After 10 years, 1.82% (11 out of 681) of patients treated with radiotherapy to the axilla had an axillary recurrence, compared to 0.93% (7 out of 744) of patients with ALDN. Distant metastases-free survival and overall survival also did not differ significantly between the arms. Second primary carcinoma was more common in patients treated with radiotherapy to the axilla compared to patients treated with ALND (75 out of 681 vs. 57 out of 744, *p* = 0.036) [36].

The results of these trials indicated that in selected patients with negative clinical findings in regional lymph nodes and positive sentinel node biopsy axillary lymph node dissection can be substituted with radiotherapy.

## 9. Patients at High Risk with Negative Findings on Axillary Surgery

Based on the results of an MA20 trial in patients with N0 disease, there is a trend to administer regional radiotherapy along with WBI in patients at high risk with negative findings on axillary surgery. In this trial, the addition of regional lymph node irradiation to WBI did not improve overall survival but decreased the recurrence rate. T3 or T4 tumors, pT2 tumors, and an insufficient number of axillary lymph nodes obtained at dissection or other risk factors, such as high grade, absence of estrogen receptor expression, and LVI are considered factors contributing to a high risk of recurrence. Regional lymph node irradiation includes supraclavicular lymph nodes and, in case axillary lymph node dissection has not been performed, also axilla. Internal mammary lymph nodes are irradiated on an individual case basis [37].

## 10. Micrometastatic Involvement of Sentinel Lymph Nodes and Axillary Irradiation

This issue was addressed by the International Breast Cancer Study Group (IBCSG 23–01) trial, in which patients with clinically negative axillary lymph nodes and positive sentinel lymph node biopsies either did or did not have axillary dissection [35]. Patients with macroscopic metastases were not enrolled. Breast-conserving surgery was performed in 91% of patients with or without axillary dissection, while the remaining patients underwent a mastectomy. The use of adjuvant radiotherapy, hormonal therapy, and chemotherapy was comparable in both arms. After a median follow-up of 5 years, the locoregional recurrence rate was 2% and 3%, respectively, and there was no difference in the disease-free survival, overall survival, or axillary recurrence rates. The results demonstrated an excellent effect of WBI. Commonly used techniques of breast irradiation also result in the delivery of substantial doses of radiation to parts of the axilla, resulting in adequate doses in patients with micrometastases. WBI without radiotherapy to the regional lymph nodes can be used as a standard of care in these patients.

Irradiation of regional lymph nodes can be offered to patients after breast-conserving surgery based on the results of the MA20 trial if risk factors are present, i.e., T3 or T4 tumors, or a T2 tumor with additional risk factors like high grade, absence of estrogen receptor expression, or LVI [37,38].

## 11. The Number of Positive Lymph Nodes and Axillary Dissection

To patients with four or more positive lymph nodes or extracapsular extension regional lymph node irradiation is offered both in patients after breast-conserving surgery and mastectomy.

In patients with one to three involved lymph nodes an individualized approach that also reflects the preferences of the patients should be selected. This strategy is based on a 2005 meta-analysis that included 8 500 lymph node-positive patients after mastectomy and axillary dissection and compared irradiation of the chest wall and regional lymph nodes with no adjuvant irradiation [1]. In patients with lymph node-positive disease, radiotherapy decreased local recurrences at 5 years from 23% to 6% (reduction of 17%), while the breast cancer mortality risk improved from 60.1% to 54.7% (reduction of 5.4%, SE 1.3, *p* = 0.0002), and overall mortality reduced by 4.4%, (SE 1.2, *p* = 0.0009). 

A 2014 meta-analysis of 1300 patients with one to three positive lymph nodes after mastectomy and axillary dissection supports the use of adjuvant radiotherapy in this subgroup. The administration of radiotherapy to the chest wall and regional lymph nodes reduced the locoregional recurrence rate from 20.3% to 3.8%, the total recurrence rate from 45.7% to 34% (RR 0.68, 95% CI 0.57–0.82), and breast cancer mortality from 50% to 42% (RR 0.8, 95% CI 0.67–0.95). However, these data should be interpreted with caution because the patients were treated between 1964 and 1986, and systemic therapy was either not administered or the regimens used are considered inferior today [39].

On the other hand, retrospective data indicate that among the patients with one to three involved lymph nodes, there may be a subgroup of patients with limited benefit from radiotherapy. A cohort of 93,793 and 36,299 patients with AJCC (The American Joint Committee on Cancer) stage oT1-2pN1 patients who underwent mastectomy were analyzed in an observational study based on the National Cancer Database (NCDB) and Surveillance, Epidemiology, and End Results (SEER) of the National Cancer Institute data. In the NCDB cohort, postmastectomy radiotherapy lowered the risk of all-cause mortality by 14% among patients with two positive lymph nodes and tumor size between 2 and 5 cm or three positive lymph nodes [hazard ratio (HR) 0.86, 95% CI, 0.81–0.91; *p* < 0.0001], but no effect was observed in patients with one or two positive lymph nodes and tumors of 2 cm or less.

The analysis of the SEER confirmed these findings. It concluded that postmastectomy radiotherapy was associated with a decrease in breast cancer-specific mortality in patients with two positive lymph nodes and tumors between 2 and 5 cm in size, or three positive lymph nodes (HR 0.86; 95% CI 0.77–0.96; *p* = 0.007), but not in patients with one or two positive lymph nodes and tumors of 2 cm or less.

The recommendations for postmastectomy radiotherapy by the joint panel of the American Societies for Clinical, Radiation, and Surgical Oncology state that postmastectomy radiotherapy lowers the risk of locoregional failure, any recurrence, and mortality in patients with T1-2 breast cancer and one to three positive axillary lymph nodes. However, in some patient subgroups, the risk of locoregional recurrence is so low that it might be outweighed by the potential toxicity. The panel agreed that clinicians should base their recommendations for individual patients on many factors. Factors that could be associated with the reduced benefit of postmastectomy radiotherapy include age of more than 40 or 45 years, reduced life expectation because of age or comorbidities or conditions that might increase the risk of complications, pathological factors associated with a low tumor volume (e.g., T1 tumors, the absence of LVI, only one positive lymph node and/or small size of lymph node metastases or a major response to systemic therapy), and biological tumor characteristics associated with improved treatment outcomes and survival and/or sensitivity to systemic treatment (e.g., low tumor grade or high sensitivity to endocrine therapy). The panel recommends directing radiotherapy to the internal mammary, supraclavicular, and axillary apical lymph nodes, in addition to the chest wall and reconstructed breast [11].

The decision regarding the indication for radiotherapy may be informed by the utilization of an online calculator designed for personalized decision-making regarding PMRT in patients with T1-2N1 breast cancer [40].

For a more precise determination of management recommendations, the results of the SUPREMO (Selective Use of Postoperative Radiotherapy after MastectOmy) trial evaluating the benefit of radiation therapy in patients after mastectomy at intermediate risk, including patients with one to three positive lymph nodes, are awaited.

## 12. Indication of Lymph Node Irradiation in Patients with 1–3 Positive Lymph Nodes

Although there is a consensus that patients with more than three positive lymph nodes should be treated with regional radiotherapy, there is controversy regarding the recommendations for patients with one to three involved lymph nodes. The current recommendation is to offer regional radiotherapy based on the results of the MA20 and EORTC trials.

The MA20 trial investigated the addition of regional lymph node irradiation to WBI in patients with positive lymph nodes or high-risk lymph node-negative patients. Axillary dissection was performed in patients with a positive sentinel lymph node. All patients were treated with adjuvant systemic therapy, including chemotherapy, endocrine therapy, or both. Among patients undergoing axillary lymph node dissection, the involvement of one to three lymph nodes was observed in 85%. The results indicated that the addition of regional lymph node irradiation to WBI after breast-conserving surgery lowered the breast cancer recurrence risk but did not improve overall survival [37].

Considering the data discussed above, the NCCN (The National Comprehensive Cancer Network) guidelines recommend consideration of radiotherapy in patients with T1-2 tumors after breast-conserving surgery and surgical axillary staging with one to three positive lymph nodes based on criteria including clinical T1–T2, clinical N0, no neoadjuvant chemotherapy, one or two positive sentinel lymph nodes, and planned breast irradiation. If all the above-mentioned criteria are met, it is recommended to consider omitting regional nodal irradiation based on a consultation with the radiation oncologist. If the criteria are not met, WBI with the irradiation of undissected parts of the axilla or regional lymph node irradiation is recommended.

In case of negative findings in axillary lymph node WBI, consideration of regional node irradiation is recommended in patients with centrally or medially located tumors, pT3 tumors, or pT2 tumors in the presence of at least one of the risk factors, including grade 3, LVI, or absence of estrogen receptor expression. In patients with the involvement of four or more lymph nodes, WBI combined with regional node irradiation and irradiation of the unresected parts of the axilla is recommended [26].

## 13. Conclusions

The current progress in the adjuvant radiotherapy of breast cancer is the result of a long-term effort, leading to improvements in therapeutic strategy by lowering the complication rate and increasing therapeutic efficacy. Radiotherapy indications have been refined based on advances in surgical techniques as well as a better understanding of tumor biology, and, most importantly, in association with technological advances in radiotherapy and the advent of new, effective drugs.

The current status of knowledge is in many respects unsatisfactory, and the results of ongoing clinical trials are eagerly awaited (Table 3). The continuation of a multidisciplinary effort integrating new diagnostic and therapeutic options remains the fundamental strategy aimed at achieving progress in the therapy of breast cancer.

## Figures and Tables

**Table 1:** Considerations for RT in patients receiving preoperative systemic therapy according to NCCN recommendations—version 1.2024 [26].

RT after preoperative therapy and breast-conserving surgery	
cN0 → ypN0 (SLNB)	WBI + boost to the tumor bed
cN+ → ypN0	WBI + boost to the tumor bed + comprehensive RNI (ongoing NSABP B-51 trial assessing the benefit of RNI)
cN+→ ypN+ (SLNB)	ALND followed by comprehensive RNI (ongoing A11202 trial assessing the benefit of ALND)
**RT after preoperative therapy and mastectomy**	
cN0 → ypN0 (SLNB)	Consider RT to the chest wall without RNI
cN+ → ypN0 (SLNB)	RT to the chest wall and comprehensive RNI
cN+ → ypN+	ALND followed by comprehensive RNI

SLNB, sentinel lymph node biopsy; ALND, axillary lymph node dissection; RT, radiation therapy; RNI, regional nodal irradiation.

**Table 2:** Considerations for RT in patients receiving preoperative systemic therapy according to the “Lucerne Toolbox” [27].

	ypN	Tx: Level I/II	RT: Level III/IV ± IM
cN0 → ycN0 → SLNB	ypN0	No local Tx	No local Tx
	ypN+	ALDR or Axillary RT	RT if risk factors *
cN+ → ycN0 → TAD	ypN0	Axillary RT	RT if risk factors *
	ypN+	ALND or Axillary RT	RT
cN+→ ycN+ → ALND	ypN0	No local Tx	RT if risk factors *
	ypN+	RT (only forgotten nodes)	RT

Risk factors: non-luminal A-like, grade 3, middle/high genomic risk, LVI, ECE. * SLNB, sentinel lymph node biopsy; ALND, axillary lymph node dissection; TAD, targeted axillary dissection; RT, radiation therapy; LVI, lymphovascular invasion; ECE, extracapsular extension; IM, internal mammary; Tx, treatment.

**Table 3:** Ongoing phase III trials whose results are awaited in adjuvant breast cancer radiotherapy.

Study Title	NCT Number	Status	Interventions
Radiotherapy after Mastectomy for Breast Cancer Patients at Increased Risk of Local Recurrence	NCT03101683 SUPREMO	Not yet recruiting	Partial Chest Wall RT
Necessity of Post-mastectomy Radiotherapy after Neoadjuvant Chemotherapy and Mastectomy	NCT05993559	Not yet recruiting	Arm I: No-PMRT Arm II: PMRT
DESCARTES: De-ESCAlation of RadioTherapy in Patients with Pathologic Complete rESponse to Neoadjuvant Systemic Therapy	NCT05416164	Not yet recruiting	Omission of RT
ALND vs. ART in Positive Sentinel Node after Neoadjuvant Therapy in Breast Cancer	NCT04889924	Recruiting	Arm I: Axillary RT Arm II: ALND
Axillary Management in Breast Cancer Patients with Needle Biopsy Proven Nodal Metastases after Neoadjuvant Chemotherapy	NCT04109079	Recruiting	Arm I: ALND Arm II: axillary RT
Eliminating Surgery or Radiotherapy after Systemic Therapy in Treating Patients with HER2 Positive or Triple Negative Breast Cancer	NCT02945579	Recruiting	Omission of RT in Exceptional Responders
Comparison of Axillary Lymph Node Dissection with Axillary Radiation for Patients with Node-Positive Breast Cancer Treated with Chemotherapy	NCT01901094 A011202	Active, not recruiting	Arm I: ALND Arm II: nodal RT Arm III: axillary RT
Standard or Comprehensive Radiation Therapy in Treating Patients with Early-Stage Breast Cancer Previously Treated with Chemotherapy and Surgery	NCT01872975 NSABP B51/ RTOG 1304	Active, not recruiting	Omission of RT
